# Contribution of STAT3 to Inflammatory and Fibrotic Diseases and Prospects for its Targeting for Treatment

**DOI:** 10.3390/ijms19082299

**Published:** 2018-08-05

**Authors:** Moses M. Kasembeli, Uddalak Bharadwaj, Prema Robinson, David J. Tweardy

**Affiliations:** 1 Department of Infectious Disease, Infection Control and Employee Health, Division of Internal Medicine, University of Texas MD Anderson Cancer Center, Houston, TX 77030, USA; MMKasembeli@mdanderson.org (M.M.K.); UBharadwaj@mdanderson.org (U.B.); PRobinson1@mdanderson.org (P.R.); 2 Department of Molecular and Cellular Oncology, University of Texas MD Anderson Cancer Center, Houston, TX 77030, USA

**Keywords:** STAT3, asthma, inflammatory bowel disease (IBD), ulcerative colitis (UC), Crohn’s disease (CD), fibrosis, cachexia

## Abstract

Signal transducer and activator of transcription (STAT) 3 plays a central role in the host response to injury. It is activated rapidly within cells by many cytokines, most notably those in the IL-6 family, leading to pro-proliferative and pro-survival programs that assist the host in regaining homeostasis. With persistent activation, however, chronic inflammation and fibrosis ensue, leading to a number of debilitating diseases. This review summarizes advances in our understanding of the role of STAT3 and its targeting in diseases marked by chronic inflammation and/or fibrosis with a focus on those with the largest unmet medical need.

## 1. Introduction

Signal transducer and activator of transcription (STAT) 3 is one of a seven-member family of proteins that transduce peptide hormone signals from the cell surface to the nucleus [1]. STAT3 is activated by over 40 peptide hormones, of which the best examined are members of the interleukin (IL)-6 family of cytokines. IL-6 is a key mediator of the acute phase response, which comprises the second wave of the acute damage response in mammals. The first wave is damage recognition, which is mediated by pathogen and damage receptor recognition of pathogen-associated molecular patterns (PAMP) produced by invading organisms and damage-associated molecular patterns (DAMP) release from damaged cells [2,3]. PAMP and DAMP recognition is mediated by several families of pathogen/damage recognition receptors, chief among which are members of the Toll-like receptor (TLR) family.

**Figure 1:**
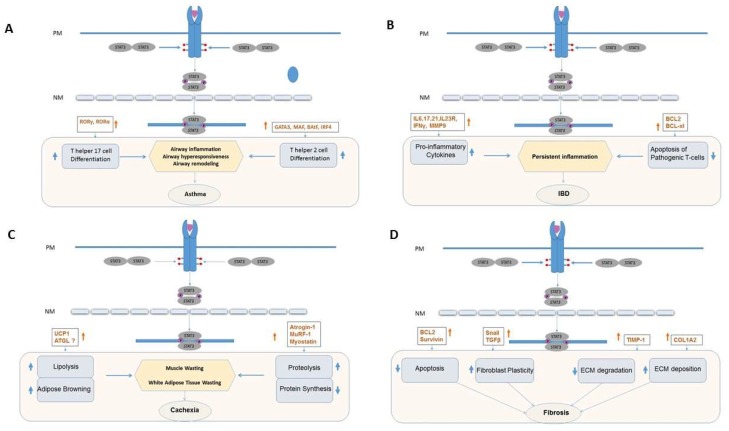
Diagram representing the pathogenic effects of aberrant STAT3 signaling in different diseases (**A**) asthma (**B**) inflammatory bowel disease (IBD) (**C**) cachexia and (**D**) fibrosis. In red are STAT3 modulated genes that contribute to the pathogenesis and progression each disease state.

TLR activation leads, in many instances, to the activation of the master regulator of the mammalian inflammatory response, NF-κB. NF-κB upregulates a variety of pro-inflammatory mediators, including TNF-α, IL-1α, and IL-6. IL-6 binding to its heterodimeric receptor composed of IL-6Rα (membrane bound or soluble) and IL-6Rβ (gp130) results in heterodimer receptor oligomerization, transphosphorylation and activation of gp130-associated Jaks, notably Jak2, which phosphorylate gp130 at tyrosine (Y) residues within its cytoplasmic tail. The Src-homology (SH) 2 domain of STAT3 binds to gp130 pY residues within the gp130 with the consensus amino acid motif, YXXQ (X is any natural amino acid.) STAT3 itself becomes phosphotyrosylated on Y705, homodimerizes through reciprocal pY-peptide/SH2 binding interactions, and accumulates in the nucleus where it binds to the promoters of genes containing specific binding elements, which leads to gene activation or repression and reprograms the cell acutely to resist apoptosis [4,5,6]. However, as the damage response persists, chronic activation of STAT3 becomes maladaptive, resulting in chronic inflammatory conditions and fibrosis. (Figure 1) This review will focus on the deleterious contribution of STAT3 to diseases marked by chronic inflammation and fibrosis and will provide examples of the beneficial effects of targeting STAT3, focusing on those diseases with the largest unmet medical need.

## 2. STAT3 and Asthma

### 2.1. Overview

Asthma affects 10% of the population worldwide and its prevalence has been increasing over the last decade [7]. It is a heterogeneous disease characterized by inflammation that leads to hyperresponsiveness of the airways and airway wall remodeling. Classically, asthma has been thought of as an inflammatory disease mediated by T helper 2 (Th2) cells and is characterized by atopy, eosinophilia, and responsiveness to steroids [8,9,10]. However, about 10% of asthma patients present a Th17 driven phenotype that is non-atopic, neutrophilic, and steroid-resistant, with higher morbidity and mortality owing to the lack of available effective treatments [11,12,13].

### 2.2. Contribution of STAT3 and Its Targeting in Asthma

Early studies indicated that STAT3 may be important for the regulation of immune cell recruitment, specifically Th2 cells during allergic inflammation. These studies demonstrated the role of STAT3 in house dust mite (HDM)-mediated allergic inflammation in which they showed that targeted deletion of STAT3 in the airway epithelium or upstream inhibition of STAT3 activation prevented HDM-mediated allergic inflammation and airway hyper-responsiveness (AHR) [14]. Subsequent studies by other labs show that STAT3 is activated during Th2 cell development and that in conjunction with STAT6, it is required for Th2 function. In the absence of STAT3, STAT6 was unable to bind to loci of genes (*Gata3*, *Maf*, *Batf*, and *Irf4*) known to drive Th2 immune related activity [15]. Interestingly, some studies suggest that Th2 cells respond to STAT3 activation differentially depending on their location in the lung. For example, STAT3-deficient T cells resident in the bronchial lymph nodes produced significantly diminished levels of Th2 cytokines, whereas, in a different model that assessed STAT3-deficient T-cells located in the airways, these cells were found to produce elevated levels of Th2 cytokines. It has been determined that these differential effects were T cell-intrinsic rather than milieu dependent [16]. However the mechanism behind the opposing role of STAT3 on Th2 cells remains to be investigated. Why previous studies had not shown similar effects with similar models is not clear, but the authors suggest that these discrepancies might be due to differences in the asthma induction protocols [16]. Together, these data demonstrate that STAT3 contributes to Th2-mediated allergic inflammation, but this contribution may be complex and varied.

The role of STAT3 in the generation of Th17 cells is well known as is its activation by several cytokines, including transforming growth factor-β (TGF-β1), IL-1β, IL-6, and IL-23, which lead to increased expression of Th17 specific transcription factors, retinoic acid-related orphan receptor gamma (RORγ) and RORα [17,18,19]. Dysregulated IL-17 production promotes the production of pro-inflammatory cytokines and chemokines which leads to the recruitment of inflammatory cells such as neutrophils to the site of inflammation [20]. Studies in mouse models show that persistent IL-17 activation leads to increased collagen deposition, airway smooth muscle mass, and mucous hypertrophy in a mouse model of asthmatic airway remodeling [21]. More recently, we demonstrated that airway inflammation and remodeling in the murine house dust mite (HDM) model of asthma is accompanied by STAT3 activation within the lung and by increased lung levels of Th2- as well as Th17-type cytokines [22].

### 2.3. Drug Targeting of STAT3 in Asthma

Pharmacological targeting of STAT3 activity by inhibiting upstream kinases has been shown to significantly reduce HDM-induced lung inflammation. This observation is in agreement with available genetic data, and strongly supports the idea that STAT3 is a crucial mediator of Th2-driven allergic responses in the lung [14]. The validity of this point of view is supported by the fact that several JAK inhibitors, e.g., TyrA1, Vr588, are currently in development (Table 1) for allergic diseases, including asthma [23]. IL-5 induced STAT3/5 signaling plays a causative role in allergic asthma and is being targeted by Mepolizumab, which proves to be quite effective in reducing risk of asthma exacerbations as well as reduce dependence on corticosteroids [24,25,26,27].

Other signaling pathways associated with asthma appear to rely on STAT3 as a downstream factor; for instance, overproduction of sphingosine-1-phosphate (S1P) has been shown to lead to chronic inflammation associated with various diseases including asthma and colitis-associated cancer through the hyper-activation of NF-κB and STAT3 [66,67]. The activation of STAT3 in mast cells by S1P has been shown to modulate early T-cell recruitment to antigen-challenged lungs through chemokine production. As a result, the administration of a S1P receptor 2 (R2) antagonist, JTE-013, attenuated inflammatory infiltration, and pretreatment with JTE-013 suppressed STAT3 activation, reduced chemokine secretion and prevented early T-cell recruitment in mice lungs after antigen challenge [29].

Direct targeting of STAT1 and STAT3 using decoy oligonucleotides (dODN) was shown to reduce airway inflammation and AHR in lungs of mice challenged with HDM [30]. The overwhelming majority of small molecules identified as inhibitors of STAT3 have been shown to target STAT3 indirectly through inhibition of tyrosine kinases upstream of STAT3 [68]. Many direct STAT3 inhibitors clearly demonstrate the ability to prevent STAT3 activation and have corroborated results of genetic targeting in preclinical models of STAT3 associated diseases; however, none has advanced to the clinic. Working with two small drug-development companies (StemMed, Ltd, Houston, USA and Tvardi Therapeutics, Inc., Houston, TX, USA), we developed an oral small-molecule STAT3 inhibitor (C188-9) that binds to STAT3 with high affinity [62] and achieves excellent exposure in cancer patients in Phase I studies. We showed that systemic administration C188-9, abrogated HDM-induced STAT3 activation, airway inflammation, and remodeling. Inhibition of HDM-induced lung changes by C188-9 was accompanied by normalization of IL-4, IL-5, IL-13, and IL-17A cytokine levels, as well as prevention of HDM-induced increases in Th2 cells, Th17 cells, and IL-4- and IL-17A-producing non-T cells [22]. Available data suggest there may be a therapeutic advantage of targeting both Th2 and Th17 in asthma to maximize therapeutic efficacy [69], which C188-9 may be able to provide.

## 3. STAT3 and Inflammatory Bowel Disease

### 3.1. Overview

IBD is an idiopathic disease of the gastrointestinal tract with an estimated prevalence of 3.1 million in the United States [70]. The two major types of IBD are ulcerative colitis (UC) and Crohn′s disease (CD) [71,72,73]. Each is distinguished by the location of inflammation within the gastrointestinal tract. In UC, inflammation is limited to the epithelial and sub-epithelial layers of the large intestine and begins in the rectum and lower colon, but may spread to involve the entire colon [74]. In CD, inflammation can extend from the mucosa to the serosa and occur in any part of the gastrointestinal tract, although the distal ileum and colon are most often affected [75,76]. Symptoms of IBD include abdominal pain, diarrhea, bloody stools, weight loss, fatigue, fever, bowel obstruction and delayed development and stunted growth in children [77,78,79]. Patients with UC and CD have a 20- to 30-fold higher risk of developing colorectal cancer (CRC) compared to the general population [80,81,82].

### 3.2. Contribution of STAT3 to IBD

The etiology of IBD is unclear, although genetic susceptibility, immune responses, environmental triggers, and the luminal microbiota have been linked to disease pathogenesis [73,83]. Genome-wide association studies have identified more than 160 loci linked to IBD susceptibility [84], including genes related to intestinal mucosal immune responses, such as STAT3 [85]. Accumulating evidence suggests that multiple cytokines play a major role in the pathogenesis of IBD [86,87]. Many of these cytokines serve as ligands for cell surface receptors that activate STAT3 [88,89]. STAT3 has been shown to be activated in actively inflamed colons from IBD patients [90]. Furthermore, increased STAT3 in T-cells, as well as macrophages and epithelial cells, has been shown to directly correlate with histological degrees of inflammation [91,92]. Genetic deletion of STAT3 in myeloid cells (neutrophils and macrophages) and enterocytes resulted in chronic colitis [93,94,95,96,97], suggesting that STAT3 activation in these two cell types, associated with innate immunity, were protective against colitis. In contrast, STAT3 activation in T-cells contributes to colitis [93,98,99]. Studies done with T-cell specific STAT3 knockout mice demonstrated attenuated T-cell proliferation [99]. Furthermore, it was demonstrated that STAT3 activation by IL-6 led to prolonged survival of pathogenic T-cells in the lamina propria [93]. Most importantly, these later studies demonstrated that blockade of IL-6 trans signaling suppressed T-cell resistance against apoptosis, which reduced intestinal inflammation [93,98], indicating that STAT3 activation in T cells contributes to colitis.

We examined the effects of genetic modulation of STAT3 in whole animals, in mouse models of UC and CD (manuscripts in preparation). Two isoforms of STAT3 (α, β), derived from one gene by alternative mRNA splicing, are expressed in most cells in a 4:1 ratio (α:β). STAT3α is pro-inflammatory and anti-apoptotic, while STAT3β has opposing effects on STAT3α. Using STAT3 Δ^β^/Δ^β^ transgenic mice, which express only the STAT3 α isoform, generated in Valeria Poli’s lab [100] we determined that manifestations of IBD, such as mortality, weight-loss, rectal and/or colonic bleeding, diarrhea, and colon shortening, were exacerbated in the transgenic mice versus cage-control WT mice [45,46]).

Several cytokines that are elevated in patients with IBD are known to activate STAT3 and or its downstream targets, including IL-6, IL-15, IL-21, IL-23, IL-17, IL-18, IL-10, IL-11, IL-22, IFNα/β /γ and matrix metallopeptidase 9 (MMP9) [101,102,103,104,105,106,107,108,109]. Pro-inflammatory cytokines within this group, such as IL-2, IL-6, IL-15, IL-21, IL-23, IL-17 and IL-18, have been demonstrated to be detrimental in mice models of IBD [93,97,110,111,112,113,114,115,116,117,118]. While anti-inflammatory cytokines within this group, such as IFNα/β, IL-10, IL-11 and IL-22, were shown to be beneficial in animal models of IBD, their administration did not show any significant beneficial effects in patients with IBD [119,120,121,122,123,124,125,126]. Our results significantly expand on those obtained previously in dextran sulfate sodium (DSS)-treated Δ^β^/Δ^β^ mice [100]. In addition to all manifestations noted by that group, our study also demonstrated significantly increased mortality in the Δ^β^/Δ^β^ mice probably due to the higher concentrations and duration of DSS that was used to induce UC, 5% DSS (for 7 days) versus 2.5% DSS (for 5 days).

### 3.3. Targeting Cytokines and Cytokine Receptors in IBD

Antibodies against some STAT3-activating cytokines and/or their receptors implicated in IBD pathogenesis in mouse IBD studies have demonstrated benefit in patients with IBD. For example, use of a humanized anti-IL6R monoclonal antibody, tocilizumab, has completed phase 2 trials in humans with CD and has shown beneficial effects [31]. Phase II studies with anti-IL-6 monoclonal antibodies in patients with active CD are underway. Two other drugs targeting IL6, TJ301 and C326, are also in trial (Table 1). Furthermore, vidofludimus, an oral immunomodulatory agent that inhibits the expression of IL17A, IL17F, and IFN-γ has shown clinical benefit in an open-label uncontrolled entrance study of patients with IBD conducted at 13 study centers in Germany, Bulgaria and Romania [34]. Additionally, IL12p40, a cytokine subunit that is shared between IL-12 and IL-23 (a cytokine responsible for maintenance of Th17 cells), has been targeted in IBD [35,36]. Significant clinical improvement of CD manifestations was demonstrated with ustekinumab, an anti-IL12p40 monoclonal antibody, within 4–6 weeks of start of treatment in two clinical trials.

### 3.4. Drug Targeting of STAT3 in IBD

A strategy that has been pursued to treat IBD is targeting of signaling pathways shared by cytokines implicated in disease pathogenesis. For example, the receptors of cytokines IL-2, IL-7, IL-9, IL-15, and IL-21 share a common subunit [127], which signals through activation of Jak3 that activates STAT3. A selective inhibitor of Jak3, Janex-1, shows promise as a therapeutic option for colitis treatment [37,38]. Preclinical studies of Janex-1 in a murine 2,4,6-trinitrobenzene sulphonic acid (TNBS)-induced colitis model demonstrated attenuation of disease manifestations [39]. Furthermore, Phase II and III clinical studies in UC patients have demonstrated benefit of tofacitinib, an oral Jak3 inhibitor, in inducing clinical responses and remissions and has been Food and Drug Administration (FDA) approved as the only nonsteroidal oral treatment that induces remission for moderate-to-severe UC [40]. In addition, filgotinib and upadacitinib (ABT-494, AbbVie), which selectively target Jak1, have demonstrated benefit in phase II clinical studies in CD patients [42].

We determined that administration of C188-9, a small-molecule direct inhibitor of STAT3 we developed (see above) was beneficial in pre-clinical models of IBD [45,46]. All manifestations of DSS-induced UC and TNBS-induced CD in mice were prevented by C188-9 treatment. C188-9 treatment also induced increased apoptosis of pathogenic CD4^+^ T-cells, and reduced colon levels of IL-17-positive cells in both models. C188-9 treatment also down-modulated levels of DSS-induced STAT3 gene transcripts involved in inflammation, apoptosis-prevention, and colorectal-cancer metastasis, while upregulating genes associated with prevention of CRC. These findings with C188-9 provide proof-of-principle that small molecule targeting of STAT3 deserves consideration as a new approach to treatment of IBD, particularly in patients refractory to current therapies.

## 4. STAT3 and Cachexia

### 4.1. Overview of Cachexia

Cachexia is a metabolic syndrome characterized by intractable muscle and fat storage wasting that is a major cause of mortality in patients with chronic low-grade inflammation such as those with end-stage heart failure, chronic kidney disease, chronic obstructive pulmonary disease (COPD), rheumatoid arthritis and cancer [128]. Cachexia is a condition that mainly affects skeletal muscle and adipose tissues as a result of an altered metabolic state in which there is a systemic negative energy balance [129,130].

Cachexia, a disease of dysregulation of metabolic homeostasis, results from impaired regulation of the balance between anabolic and the catabolic states leading to loss of fat and skeletal muscle mass. Patients present with signs of metabolic dysfunction, such as increased insulin and Insulin-Like Growth Factor-I (IGF-1) resistance, induction of mitochondrial uncoupling proteins and fat tissue browning [130]. Maintenance of tissue protein content is a tightly regulated process involving the balanced control of protein synthesis and protein degradation. Loss of muscle mass is mainly attributed to increased proteolysis and suppressed protein synthesis. Some drivers of protein turnover associated with cachexia have been extensively characterized, for example, the IGF1/PI3K/Akt pathway is known to regulate skeletal muscle mass by promoting protein synthesis and inhibiting muscle protein degradation, through mTOR and forkhead box (FOXO)s signaling, respectively [131]. On the other hand, classical catabolic signaling, such as the myostatin–Smad3 pathway, acts to negatively regulate muscle mass by inhibiting AKT activity, which suppresses protein synthesis while promoting ubiquitination and protein degradation via muscle-specific ubiquitin ligases muscle atrophy F-box (MAFbx) and muscle RING Finger-1 (MuRF1) and the ubiquitin proteasome system (UPS) [132]. These events are usually tightly balanced through a homeostatic process that is responsive to external stimuli, including nutrients, hormones and cytokines. While, acute insults to homeostasis are usually countered by controlled inflammatory responses [133,134], it is hypothesized that chronic inflammation leads to an imbalance that manifests as a disease state, in this case cachexia.

### 4.2. Inflammation and Cachexia

Although factors contributing to cachexia are complex and multifactorial, Inflammation appears to be a central aspect underlying its pathophysiology [135]. Several studies in rodent models and patients show a close association between cachexia and systemic chronic inflammation. Pro-inflammatory cytokines, such as TNF-α, IL-6, and IFN-γ, have been shown to be major drivers of cachexia as they promote tissue proteolysis and increase adipose tissue browning [130,136,137]. The catabolic effects of proinflammatory cytokines have been shown to accelerate protein turnover rates by upregulating proteolytic pathways such as the proteosomal degradation system (UPS) and autophagy [138]. Accumulating evidence demonstrates molecular connections between pro-inflammatory cytokines and uncontrollable protein and lipid catabolism. For instance, TNF-α inhibits myocyte differentiation through NF-κB activation in cultured C2C12 muscle cells. In addition, muscle-specific transgenic expression of activated κB kinase β (MIKK) has been shown to cause significant muscle wasting due to increased expression of the E3 ligase, MuRF1 [139]. TNF-like weak inducer of apoptosis (TWEAK), with its cognate receptor FN14 have also been implicated in muscle wasting and have been considered candidate targets for therapeutic intervention [140], although drugs targeting TNF-α have had limited success in cachexia human trials [141].

### 4.3. IL-6, JAK and STAT3 Signaling Axis in Cachexia

Despite some conflicting studies in mice concerning a direct role of IL-6 in cachexia [142], the preponderance of evidence shows a strong link between IL-6 and diseases associated with cachexia, including chronic infections, chronic kidney disease (CKD), sepsis, and cancer. Accumulating evidence suggests that IL-6 and other members of the IL-6 family that activate STAT3, such as IL-11, leukemia inhibitory factor, oncostatin M and ciliary neurotrophic factor, play an important role in the development and progression of cachexia and have increasingly been implicated in the molecular mechanisms of muscle atrophy.

Results from early experiments demonstrated that muscles of mice that transgenically overexpressed IL-6 displayed a significant loss of muscle mass akin to cachexia. Inhibition of IL-6 activity using anti–mouse IL-6 receptor (mIL-6R) antibody reversed all the changes associated with atrophy [143]. Findings from the Apc (Min/+) cachexia mouse model suggest that systemic IL-6 is necessary for adipose and skeletal muscle atrophy [144]. Additional data obtained in the same mouse model clearly demonstrated that ATP dependent proteolysis is linked to IL-6 signaling [145]. Data from patient studies further solidify the notion that IL-6 blockade mitigates cachexia. For example, the IL-6 inhibitor, tocilizumab, has shown promising results in the treatment of cancer cachexia. In addition, phase I and II trials of the humanized anti-IL-6 antibody, ALD518, were shown to ameliorate lung cancer-related anemia and cachexia [146]. Overall, these studies show that the IL-6 signaling plays a major role in the development and progression of cachexia and contributes to crucial aspects of the disease such as loss of muscle mass and fat atrophy.

Numerous functional and mechanistic studies have been done to elucidate the link between muscle and adipose tissue atrophy and IL-6 signaling. These studies have shown a strong association between phosphorylation of STAT3 at Y705 and muscle atrophy. It has been established in various mouse models of cachexia that IL-6 catabolic signaling in cachexia is exerted through the transcription factor STAT3 [145,147].

### 4.4. STAT3, Proteolysis, and Activation of the Ubiquitin Proteosome System (UPS)

IL-6-mediated cachexia has been shown to be associated with increased proteolytic activity in skeletal muscle cells. Recent studies have uncovered how STAT3 signaling engages the muscle specific proteolytic pathways in cachexia to induce muscle mass loss. Insights into how STAT3 activation leads to protein degradation in cachexia came from studies that looked into the effects of catabolic conditions such as CKD and streptozotozin-induced acute diabetes on muscle metabolism in muscle-specific STAT3 knock out mouse [48]. The data from these mouse models showed that specific knockout of STAT3 in muscles or pharmacological inhibition of STAT3 significantly reduced muscle wasting. Further analysis revealed that CKD induced STAT3 phosphorylation at Y705, leading to increased expression of C/EBPδ and the transcriptional regulation of myostatin expression [48]. This process then leads to the activation of caspase-3 and the UPS. The role of STAT3 in protein muscle catabolism was further elucidated in a subsequent study using several mouse models of cancer cachexia. These studies showed that JAK/STAT3 signaling activates two distinct proteolytic pathways. The first involves activation of caspase 3 transcription by direct binding of STAT3 to caspase-3 promoter sites in muscle cells. Caspase-3 proteolytic activity on actinomyosin and the myofibrillar proteins has been shown to be the precursor step in muscle breakdown via UPS. The second mechanism is through pY-STAT3 transcriptional activation of CCAAT-enhancer-binding proteins (C/EBPδ), which upregulates myostatin, a negative regulator of protein muscle mass that is known to increase the expression of muscle specific E3 ligases MAFbx and Atrogin-1 leading to protein degradation via the UPS.

### 4.5. STAT3 in Autophagy-Mediated Muscle Loss

Autophagy is another potential mechanism by which muscle mass is modulated in cachexia. Autophagy is an evolutionarily conserved process that is deployed under stress as a survival mechanism to maintain homeostasis. The role of autophagy in maintenance of proper muscle function is complex. Basal levels of autophagic activity may be necessary for the maintenance of muscle mass, as evidenced by data in mice with muscle specific deletion of a gene required for autophagy, autophagy related 7 (ATG7); these mice suffer significant loss of muscle mass. In fact, diminished autophagy has been linked to certain forms of muscle myopathies [148]. On the other hand, recent data show that autophagy is induced in skeletal muscles of cancer patients with cachexia [149]. Indeed, using three different mouse models of cancer cachexia, Penna et al. definitively show that autophagy is significantly induced in muscles of these tumor-bearing mice and contributes to muscle wasting [150].

The role of STAT3 in autophagy is equally complex and varied, as STAT3 has both anti and pro-autophagy roles depending on the context [151]. Recent data suggest that IL-6 binding to gp130 receptor is a potent inducer of autophagy in C1C12 myotubes [152]. However, this effect was only specific for myotubes as opposed to myoblasts, despite detection of elevated levels of pY-STAT3 in both.

### 4.6. STAT3 in Lipolysis and Adipose Tissue Browning

Rapid loss of white adipose tissue (WAT) is a defining feature of cachexia and usually precedes muscle wasting. In addition to white adipose tissue depletion, cachectic patients also gain brown adipose tissue (BAT) that is rich in mitochondria that are uncoupled from ATP production [153]. This process further contributes to negative energy and the lipid balance characteristic of cachexia. There is evidence to suggest that IL-6 might promote cancer cachexia by regulating WAT lipolysis in early-stage cachexia and BAT generation in late-stage cachexia. For example, anti-IL-6 receptor antibody treatment inhibited WAT lipolysis and BAT generation in cachectic mice [154]. Expression of constitutively active STAT3 (C-STAT3) has been shown to restore BAT differentiation of Tyk2−/− preadipocytes and reverse the obese phenotype in Tyk2−/− mice [155]. A recent report showed that transient activation of the JAK family of kinases and STAT3 signaling in the context of β-adrenergic tissue remodeling increased the potential for thermogenic adipocyte differentiation i.e., browning [156]. Although these data suggest that there is a role for STAT3 in adipose tissue browning, the authors also point-out that this is only in the case of transient inflammation but not chronic inflammation. Therefore, it still remains a subject of further study whether STAT3 actually promotes adipose tissue browning in cachexia and, if so, what mechanisms are involved.

Lipid homeostasis is maintained through the balanced control of lypolytic and lypogenic pathways, which are subject to external modulatory factors such as hormones and cytokines. For example, Jak2 seems to play an important role in adipose tissue catabolism as shown by bodyweight gain due to adiposity in Jak2 adipose specific knockout mice [157]. The increased rate of adipose tissue breakdown associated with cachexia has been ascribed to increased expression of two major lipases–adipose tissue triglyceride lipase (ATGL) and hormone sensitive lipase (HSL). Under cachexia-inducing conditions, ATGL knockout mice show preservation of lipid mass, as well as diminished muscle mass loss, indicating that ATGL activity contributes to tissue loss in both compartments. Studies in the C26 cancer cachexia mouse model suggested an association between elevated adipose tissue mobilization and increased STAT3 phosphorylation that also coincided with increased ATGL expression levels [158]. Mice lacking the gene for STAT3 in adipose tissue appear to have more weight and increased adipose tissue mass due to hypertrophy, suggestive of inhibited lipolysis [159,160]. Others have found an association between leptin-induced lipolysis, ATGL induction, and STAT3 signaling in bovine adipocytes [161].

### 4.7. Drug Targeting of STAT3 in Cachexia

As discussed above, available data identify STAT3 as a target for cachexia intervention. Several known STAT3 inhibitors, designed to directly target hyper-phosphorylated STAT3 in cells, have been used as chemical probes to corroborate genetic experiments that highlight the role of STAT3 in cachexia. A majority of compounds that show inhibitory activity toward STAT3 activation also reduced markers of cachexia in cell culture models. For instance, treatment of C2C12 myotubes for 48 hours with a cell-permeable STAT3 SH2 domain mimetic peptide (SIP) resulted in modest myofiber hypertrophy and prevented IL-6-induced fiber atrophy [47].

We have shown that treatment of the CDK cachexia mouse model with the small molecule STAT3 inhibitor, C188-9, antagonized catabolic signaling by decreasing myostatin expression and the activation of its downstream signaling mediators, p-Smad2 and p-Smad3 [48]. In addition, C188-9 increased muscle mass in tumor-bearing mice by augmenting muscle protein synthesis and suppressing protein degradation. Mechanistic studies showed that inhibition of p-STAT3 within the mouse cancer cachexia model with C188-9 treatment reduced transcript levels of muscle-specific ubiquitin ligases MAFbx/Atrogin-1 and MuRF1 and improved protein synthesis in muscles of mice bearing tumors [147]. In addition to blocking muscle atrophy, there are data to suggest that STAT3 inhibitors can also inhibit lipolysis, for example, incubation of bovine adipocytes with the covalent inhibitor of STAT3, Stattic, reduces the levels of ATGL [162], supporting the idea that STAT3 is also involved in the regulation of white adipose tissue content.

Other modalities of STAT3 inhibition have also been investigated in cachexia with promising results. For example, there are reports that the histone deacetylase (HDAC) inhibitor, AR-42, is a potent inhibitor of STAT3 activity [49]. Recent experiments in the C26 cachexia mouse model using anabolic androgen therapy in combination with HDAC inhibitor AR-42 was shown to block STAT3 mediated muscle atrophy [163]. Ruxolitinib, a Jak1/2 inhibitor has also been found to result in a significant increase in body weight, in a phase II trial, in exocrinemetastatic pancreatic cancer patients [50,51]. Overall, these findings show that pharmacological inhibition of STAT3 activity mirrors the results obtained with genetic targeting of STAT3 and support the concept that STAT3 is a valid target for cachexia treatment.

## 5. STAT3 and Fibrosis

### 5.1. Overview of Fibrosis

Tissue fibrosis is a pathological condition that affects almost every organ in the body. It imposes a significant worldwide disease burden with very few effective therapies available. Estimates show that in developed countries alone, approximately 45–50% of deaths are attributable to fibrosis [164,165]. Fibrosis can be regarded as the unchecked process of wound healing after injury [164]. Mammalian wound healing and tissue repair involve the rapid synthesis and deposition of the extracellular matrix (ECM) to maintain tissue integrity. Wound healing is a complex process involving a series of intricately orchestrated biological processes including cell proliferation, remodeling of the extracellular matrix, cell invasion and migration. Under normal conditions it is a highly regulated process and self-limiting. However, when dysregulated, the process leads to fibrosis, which is characterized by the overproduction of the extracellular matrix and excess matrix contraction [166].

Myofibroblasts have long been identified as the chief culprit in fibrosis development. The persistent expansion and activation of tissue myofibroblasts is a salient feature of fibrosis and a major driver of disease progression [167]. Myofibroblasts are responsible for the deposition and maintenance of the fibrotic extracellular matrix (ECM) in all organs, which ultimately leads to deleterious tissue architectural changes and organ failure [168]. Acute and chronic inflammation has been associated with uncontrolled responses to tissue injury, which leads to high concentration of both endocrine and paracrine factors that stimulate the trans-differentiation of organ resident precursor cells, such as fibroblasts or pericytes, into ECM-generating myofibroblasts [167,169]. It has also been proposed that myofibroblasts arise from mesenchymal stem cells that migrate from the bone barrow [170].

Because improper regenerative healing is closely linked to inflammation, anti-fibrosis strategies have traditionally focused on inhibiting upstream pro-fibrotic cytokines and growth factors with the aim of blocking persistent myofibroblast generation, expansion and activation. Several important signaling pathways have been identified as frequently activated in fibrosis, including the Transforming Growth Factor (TGF)-β, Wingless/INT (WNT) and Yes-associated protein (YAP)/ transcriptional coactivator with PDZ-binding motif (TAZ) components of the hippo signaling pathway [171]. TGF-β signaling is a prominent player in the development of fibrosis. It is known to induce differentiation of fibroblasts to myofibroblasts and stimulate the production of ECM components. Consequently, it has attracted considerable interest as a potential target for anti-fibrotic therapy especially its downstream canonical signaling components [172,173]. This approach, however, has proven ineffective, perhaps due to the redundant nature of pro-fibrotic cytokine signaling. In addition, the clinical outcomes do not necessarily reflect the promising results that have been observed in animal studies suggesting, perhaps, that the disease models are imperfect [171,173]. This has prompted a search for new approaches to counter fibrosis. In this section of the review, we explore the emerging role of STAT3 in the pathophysiology of fibrosis in various organs.

### 5.2. Contribution of STAT3 to Fibrosis

STAT3 activation during tissue repair has been extensively studied because of its prominent role following tissue injury. STAT3 is activated as a result of the inflammatory reaction that constitutes the first stage of wound healing [174]. Moreover, It has been shown to be involved in the subsequent stage of wound repair, as observed in the epidermis-specific deletion of STAT3, which impedes epithelial repair after wounding, suggesting that STAT3 is important for re-epithelialization that occurs during wound repair [175].

It is known that under normal conditions STAT3 plays a positive role in cell survival and proliferation. However, it has deleterious effects when persistently activated, contributing to a wide variety of pathological conditions [88]. Similarly, a well-regulated process of wound healing and tissue repair is necessary for the survival of many multicellular organisms. However, when the process continues unchecked, it leads to fibrosis. Given its well-documented role in tissue injury and repair, it is not surprising that persistent activation of STAT3 might play a crucial role in fibrosis. Activation of STAT3 has been detected in many fibrotic tissues [176]. However, the molecular mechanisms that explain the role of STAT3 in the initiation and progression of fibrosis are still not fully understood. The role of STAT3 in fibrosis generally mirrors its role in other pathological conditions in which it is persistently activated. For example, in carcinogenesis, STAT3 has a prominent role in cell proliferation, prevention of apoptosis, induction of angiogenesis and plays a role in conferring cell plasticity, all of which are cellular process involved in the development and progression of fibrosis.

### 5.3. STAT3 and the ECM

Several studies show that STAT3 contributes to fibrosis by inducing the production of the ECM. Collagen type I (COL1), which consists of two α1 (COL1A1) chains and one α2 chain (COL1A2), is a hallmark of fibrosis. TGF-β through its downstream effectors has a major influence on the production of the ECM and is by far the predominant signaling network driving fibrosis. Several non-canonical transcription factors have been shown to bind the COL1A2 gene promoter to enhance its transcription in response to TGF-β stimulation, including STAT3. Recent findings demonstrate that, in conjunction with JunB, STAT3 is able to directly control COL1A2 enhancer activation [177]. Similar observations have been made in other models of fibrosis that attribute increased TGF-β1 and the consequent collagen I production, to STAT3. For instance, in intestinal mesenchymal muscle cells of patients with Montreal B2 fibrostenotic Crohn′s disease, phosphorylation of STAT3 at S727 downstream of TGF-β was shown to contribute to the fibrotic element of the disease [178]. Moreover, in fibrotic kidney cells, inhibition of the Fyn/STAT3 pathway attenuated the expression of type I collagen, fibronectin, α-smooth muscle actin, and plasminogen activator inhibitor-1 all independently of SMAD3 [179]. These findings support the notion that STAT3 plays a crucial role as a modulator of fibroblast extracellular matrix remodeling and contributes to the dysregulation of ECM deposition during fibrosis.

Another mechanism for STAT3 modulation of fibrosis is through the transcriptional control of matrix metalloproteinases (MMPs) and their cognate inhibitor proteins—tissue inhibitors of metalloproteinases (TIMPs)—that are important in the maintenance of the ECM. Although MMPs are mainly known to mediate degradation of the extracellular matrix and hence, thought to be anti-fibrotic, they have also been linked to a variety of other cellular processes that modulate fibrosis [180]. In fact, some members of this family of proteins have been shown to be pro-fibrotic. A good example is MMP-9 that promotes renal fibrosis and epithelial-mesenchymal transition (EMT) during obstructive renal injury [181]. Studies on renal fibrosis by Matsui et. al. in a unilateral ureteral obstruction (UUO) model suggest that STAT3 activation contributes to MMP-9 up-regulation and tubulointerstitial fibrosis during kidney obstruction [182].

On the other hand, TIMP-1 is an important player in ECM maintenance that binds to and inhibits activity of MMPs. It is a known downstream gene target of STAT3 [183] and plays an important role in protecting against acute and chronic liver injury as it has been shown to inhibit liver fibrosis induced by CCl_4_. In addition, the levels of TIMP-1 in the liver and serum after chronic CCl_4_ treatment were markedly diminished in hepatocyte-specific STAT3 knockout mice [184].

### 5.4. STAT3 and Fibroblast Apoptosis

Apoptosis of ECM-producing cells also is important for resolution of the wound-healing process. Because STAT3 is known to confer strong proliferative effects and resistance to apoptosis [185], it is thought to contribute to the proliferation of myofibroblasts and their persistence, leading to the characteristic accumulation of connective tissue seen in fibrosis. In lung fibroblasts from patient with idiopathic pulmonary fibrosis (IPF), IL-6 was shown to induce resistance to FAS ligand-mediated apoptosis; however, normal lung fibroblasts were sensitive to FAS ligand-induced apoptosis [186]. Interestingly, these opposing outcomes were both found to be STAT3 dependent, suggesting a context-dependent component of the STAT3 effect. These observations are supported by numerous other studies that clearly establish the anti-apoptotic role of STAT3 in fibrosis [52,187,188].

### 5.5. STAT3 and Fibroblast Plasticity

Understanding the molecular mechanisms behind the biogenesis of ECM-producing myofibroblasts is thought to be of great importance and has powerful implications in the development of effective anti-fibrotic treatments. There still is an ongoing debate regarding the lineage of myofibroblasts found in fibrotic tissues. Based on genetic lineage tracing, ECM-producing myofibroblasts in almost all organ systems appear to arise from a diverse origin involving organ resident cells such as, mesenchymal stem cell (MSC), fibroblast and pericytes. The transition to myofibroblast occurs in response to numerous growth factors and cytokines, predominantly TGFβ and others including WNT, epidermal growth factor (EGF), and IL-6 that are locally produced [166,171]. These networks have been shown to engage STAT3 as a downstream factor to generate ECM-producing cells by induction of differentiation of organ resident cells via canonical and non-canonical pathways. Several lines of evidence point to the fundamental role of STAT3 in fibroblast plasticity during fibrosis. We have shown that the IL-6 family of cytokines, which signal through STAT3, may also contribute to lung fibrosis mouse models of fibrosis and that pharmacological inhibition of STAT3 decreased fibrosis in these models [52,63].

Although data are emerging that challenge the idea that some myofibroblasts have an epithelial lineage, it still remains a widely held view that mesenchymal cells arising from aberrant epithelial-to-mesenchymal transition (EMT) contribute significantly to the population of myofibroblasts in fibrosis. STAT3 is known to play an important role in EMT. For example, kinases such as Fyn and Src have been known to activate STAT3 and drive EMT in many tumor systems [189,190]. Interestingly, fibronectin, a component of the extracellular matrix, has been shown to induce EMT in breast cancer models via the activation of STAT3 [191]. Cells harboring oncogenic RAS respond to TGF-β treatment by activation of STAT3, which in turn enhances the induction of snail, a well-known driver of EMT in fibrosis as well as metastasis [192]. Importantly, in a renal fibrosis model, SMAD3 and STAT3 have been demonstrated to act downstream of TGFβ1 to induce EMT of renal tubular epithelial cells (TECs). This observation was made when cells obtained from mice subjected to UUO were treated with TGF-β, which led to increased levels of SMAD3 and STAT3 phosphorylation and concomitant activation of snail gene expression [193,194]. Recently, other researchers and ourselves have shown in mouse models of skin fibrosis that TGF-β phosphorylated STAT3 in a non-canonical fashion and that inhibition of STAT3 activity prevented TGFβ-induced fibroblast-to-myofibroblast transition [64,195].

### 5.6. Interplay between STAT3, TGF-β1 and Other Signaling Networks

STAT3 and TGF-β1 are involved in a complex interplay that appears to have broad functions in different biological contexts. Data on the effects of STAT3 activation on TGF-β signaling are not always straightforward and, at times, are paradoxical. For example, most studies suggest that STAT3 activation drives the induction of TGF-β1 and is one of the mechanisms by which STAT3 enhances organ fibrosis. Another study by O′Reilly et al also showed that STAT3 activation by IL-6 in dermal fibroblasts leads to TGFβ activation through an indirect mechanism mediated by Gremlin-1 [196]. However, there are some data that contradict this observation, albeit in different cell systems. For instance, in vitro experiments in HeCaT cells suggest that STAT3 activation attenuates TGF-β1 via Smad3-STAT3 interplay [197]. Nevertheless, an overwhelming majority of data clearly show that STAT3 is frequently activated in almost all fibrotic systems and the STAT3/ TGF-β pathway may be engaged in a complex feedback loop. There also is a large amount of data showing that TGF-β1 signaling induces phosphorylation and activation of kinases that are known to activate STAT3. The complex nature of these signaling programs further suggests that STAT3 positively modulates fibrosis signaling by diverse mechanisms.

### 5.7. Drug Targeting STAT3 to Treat Fibrosis

Blocking STAT3 activity can be accomplished indirectly through targeting of upstream components of JAK/STAT signaling network, which can be problematic because of the redundant and adaptive nature of the fibrotic signaling [198], or directly using small molecule inhibitors, which target a single point of signal convergence in fibrosis. In rats administered JSI-124, a Jak2 inhibitor that targets STAT3 indirectly, several markers of bleomycin-induced lung fibrosis were reduced [52]. Several other Jak inhibitors, e.g., Cucurbitacin B, Pacritinib and Ruxolitinib, are at various stages of preclinical and clinical development (Table 1). Another indirect approach to STAT3 inhibition involves the up-regulation/activation of natural STAT3 antagonists. For instance, peroxisome proliferator-activated receptor gamma (PPARγ) has been shown to bind STAT3 as well as Smad3 and inhibit TGFβ1-induced phosphorylation and nuclear translocation of both molecules. [199,200]. Treatment of fibroblasts with the PPARγ agonist, rosiglitazone, highlights potential alternative avenues to modulating profibrotic signaling, as it has been shown to significantly attenuate TGFβ1-mediated up-regulation of fibrotic markers Alpha-Actin-2 (ACTA2) and COL1A1 [201].

We tested our small molecule inhibitor of STAT3, C188-9, in several animal models of fibrosis [62,65]. In an intraperitoneal bleomycin lung fibrosis model, we showed that inhibition of STAT3 phosphorylation by C188-9 ameliorated the development of pulmonary fibrosis in mice exposed to bleomycin. C188-9 also reduced expression of genes associated with type II alveolar epithelial cell (AEC) injury and fibrosis. Additionally, myofibroblast differentiation was mitigated [63].

C188-9 also showed efficacy in two models of scleroderma—the subcutaneous bleomycin (BLM) model and the tight-skin mouse (Tsk-1) model. Reduction in pY-STAT3 levels by C188-9 reduced fibrosis in both models as measured by loss of collagen accumulation and decrease in dermal and hypodermal thickness in each respective model [64]. Similar to the pulmonary fibrosis model discussed above, myofibroblast differentiation was significantly attenuated, and molecular markers of fibrosis including COL1A1, α-SMA, TGF-β, CTGF, IL-6, fibronectin, and Cad11 were diminished in response to C188-9 administration. Most importantly, mice in all models that received C188-9 showed minimal signs of toxicity. Another STAT3 inhibitor S3I–201 not only impaired the progression of fibrosis in a bleomycin model of scleroderma but also appeared to induce regression of established fibrosis [195]. In addition, inhibition of STAT3 with S3I–201 has been evaluated in a preclinical animal mouse model of renal interstitial fibrosis induced by unilateral ureteral obstruction [60]. This compound attenuated interstitial fibrosis and showed a fibrotic suppression profile that was similar to all the other inhibitors described in this section.

In the hepatic carbon tetrachloride (CCl_4_) model of fibrosis, cucurbitacin-B mitigates fibrosis through inhibition of STAT3. STAT3 inhibition coincided with diminished levels of hydroxyproline in liver tissue as well as expression of collagen-1α, α-SMA and TGF-β [54]. STAT3 inhibitor HJC0123 suppressed fibrotic markers in hepatic stellate cells; however, the study did not show data in animal models [202]. We have shown that C188-9 administration to hepatocyte-specific *Pten* knockout (Hep*Pten*-) mice, a mouse model of nonalcoholic steatohepatitis (NASH), reduced liver steatosis and hepatic fibrosis in addition to blocking progression of hepatocellular carcinoma [65]. Another direct STAT3 inhibitor, S3I–201 (Table 1) has also shown attenuation of fibrosis in preclinical models [60,61].

In summary, an abundance of evidence supports the hypothesis that STAT3 makes critical contributions to the development and progression of fibrosis in many organ systems. These studies also demonstrate that use of STAT3 inhibitors in preclinical animal modes shows promising anti-fibrotic activity that strongly suggests that STAT3 is a bona fide target for treatment of fibrotic diseases in patients.

## 6. STAT3 and Integration of Complex Signaling Networks

Considering that most of the diseases described in this review occur in an inflammatory background, an environment in which cells are exposed simultaneously to a large number of different stimuli, it would be logical to conclude that disease phenotypes arises from the integration of more than one signaling pathway [171]. The emerging view now is that STAT3 plays the role of a signal integrator; this hypothesis is supported by the fact that STAT3 activity is modulated via myriad posttranslational modifications including tyrosine and serine phosphorylation, lysine acetylation, and lysine and arginine methylation and can respond to a wide variety of stimuli also shared by other signaling networks [203].

Multiple posttranslational modifications confer STAT3 with signal integration potential, which may act in a manner akin to electronic logic gates to determine the biological outcomes of combinatorial signaling processes. Complex signaling processes elicited by chronic inflammation appear to coincidentally activate unique sets of pathways in which information such as timing of the signals and signal intensities and their localization are integrated to determine phenotypic outcomes. Indeed, Waitkus et al show that STAT3 functions as a cellular coincidence detector to enhance IEG expression after simultaneous EGFR/ protease-activated receptor-1 (PAR1) activation and is a critical point at which signals from both pathways converge [204]. Furthermore, they show in subsequent studies the role of post-translational modifications of STAT3 in signal integration, where they describe a functionally distinct non-canonical phosphorylated STAT3 protein that mediates signal integration and gene expression following EGFR/PAR1 activation [203].

Another example of the role of STAT3 as an integrator can be seen in a phenomenon referred to as ′TGF-β switch′ in which TGF-β appears to have tumor suppressive effects in epithelial cells, whereas in tumor cells it is pro-tumorigenic. Other data show that K-ras mutations alter the STAT3 interactome leading to the switch that induces non-canonical STAT3-mediated production of Snail, leading to EMT and tumor progression [192].

Recent work by Chakraborty et al. provides concrete evidence that STAT3 integrates TGF-β signaling in fibrotic skin cells. They show that STAT3 is activated by various kinases, including JAK, Src, c-ABL, and JNK, in response to TGF-β stimulation and that genetic and biochemical blockade of STAT3 activity prevented TGF-β-induced fibroblast-to-myofibroblast transition and mitigated skin fibrosis in two mouse models of scleroderma [195]. In addition, it appears that this phenomenon is not limited to TGF-β signaling, as STAT3 also facilitates other known fibrotic signaling networks. Salvador 1 (SAV1), for example, is a scaffolding kinase responsible for the cytoplasmic sequestration of pro-fibrotic transcriptional factor YAP. Leunga et al. observed increased STAT3 activation in interstitial fibrosis resulting from loss of SAV1 [205]. Importantly, chemical inhibition of YAP mediated fibrosis led to loss of STAT3 activation, pointing to a possible link between STAT3 activation and Hippo network-mediated signaling in fibrosis. The convergence on STAT3 of many of the non-canonical signaling pathways associated with fibrosis makes it an attractive therapeutic target for organ fibrosis.

There also are examples of STAT3 serving as a signal integrator in other inflammatory diseases. For example, the collaboration between STAT3 and NF-κB pathways has been shown to modulate IFN-γ/TNF-α-induced muscle wasting, suggesting that inflammatory signaling acts through integrated networks of downstream effectors, such as STAT3 and NF-κB to induce cachexia [206].

Crosstalk between multiple signaling networks is integral to the pathogenesis and progression of many inflammatory associated diseases. The concept of STAT3 as a signal integrator can help in understanding the role that it plays in a broader context, especially as a crucial mediator of a wide variety of inflammatory diseases.

## 7. Outlook

These are frustrating times for patients with chronic inflammatory diseases refractory to current treatments, such as steroid-resistant asthma, or those with diseases for which no truly effective treatments exist, including patients with cachexia and fibrosis. The evidence reviewed above unequivocally implicates STAT3 in each of these diseases. Strategies aimed at IL-6, its receptor, or members of the JAK kinase family, all of which target STAT3 indirectly, show promise. There is hope that one or more of these strategies may lead to a treatment that becomes standard of care. However, based on the findings for C188-9, there is reason for even greater hope of developing a safe and broadly effective agent that directly targets STAT3 to treat these diseases.

As the number of STAT3 inhibitors in development increase and some are tested in clinical trials, there are potential pitfalls to be considered in the clinical development of these agents. For instance, most STAT3 inhibitors have been designed to target protein/protein or protein/DNA interactions, which pose considerable challenges with respect to specificity, considering that there are close structural similarities among STAT proteins. It is not surprising that many STAT3 inhibitors suffer from lack of specificity [62,68]. Whatever impact this will have on the development and advancement of these inhibitors moving forward remains to be determined.

In addition, because of the important role that STAT3 plays in normal cell function, for example, in wound healing and Th17-related immune defense, there is a potential risk of impaired wound healing and immunosuppression that needs to be taken into account during clinical trials and in Phase IV evaluations, should an agent be FDA-approved. Presently, some STAT3 inhibitors have advanced into Phase I/II/III studies. Thus far, negative impacts on wound healing and immune function have not been reported. Clearly, further study is necessary to definitively determine the full impact of STAT3 inhibition, including any adverse effects that may arise.

Another area of interest with regard to STAT3 inhibition involves its well-known role in mitochondrial function; STAT3 is required for optimal function of the electron transport chain [207]. Thus, there is a potential risk of toxicity in the use of STAT3 inhibitors associated with inhibiting mitochondrial function, which may be unacceptable, especially in non-cancer indications, such as inflammation and fibrosis. Because mitochondrial dysfunction is increasingly implicated in drug-associated toxicities, there is mounting interest in examining the potential effect of STAT3 inhibitors on mitochondrial function and its implication for drug-related toxicities. Recent work by Genini et al. indicated that some STAT3 inhibitors, including OPB-51602, inhibit mitochondrial function, which helps to explain the lactic acidosis and peripheral neuropathy observed in some Phase I patients and poses obstacles to further clinical development of this agent [208]. Of note, preclinical and clinical data to date suggest that some direct, small-molecule STAT3 inhibitors, such as C188-9, however, do not affect mitochondrial function and these toxicities may be avoided.

## Figures and Tables

**Table 1:** STAT3 inhibitors at various stages of pre-clinical and/or clinical testing for Asthma, IBD, cachexia and Fibrosis.

Inhibitor	Target	Preclinical/Clinical Model	Goals/Results	Ref.
**Asthma**				
**TyrA1**	Jak	HDM-induced STAT3-mediated mice model of asthma	Blocked HDM-induced STAT3 activation and airway eosinophilia in mice	[14]
**Vr588**	Jak	HDM-induced STAT3-mediated mice model of asthma. Intra-nasal VR588 (1.5 and 50 mg/kg) vs oral 15 mg/kg PK. HDM extract (25 μg) intranasally 5 days/week for 3 weeks with multiple intranasal doses (1.5 to7.5 mg/kg) given 1 hour prior to each HDM exposure; a separate group administered a VR588 7.5 mg/kg intranasal dose only during the last week of HDM treatment (*n* = 8). Comparison was made with oral tofacitinib (15 mg/kg) and fluticasone propionate (1.5 mg/kg).	VR588 resulted in significant reduction of AHR at least comparable to that achieved by FP. All VR588 doses significantly reduced BAL total cell count with a variety of doses inhibiting macrophage, neutrophil, lymphocyte and eosinophil counts. VR588 attenuated the induction of numerous cytokines (IL-4, IL-5, IL-17) compared with saline control, As well as HDM induced pSTAT3	[27,28]
**Mepolizumab**	IL5 antagonist	Rhinovirus-induced Allergic Asthma Exacerbations; multicenter, double-blind, placebo-controlled DREAM trial	Mepolizumab is an effective and well tolerated treatment that reduces the risk of asthma exacerbations in patients with severe eosinophilic asthma	[24,25,26,27]
**JTE-013**	S1PR2	Dinitrophenyl (DNP) induced asthma model	Suppressed STAT3 activation, reduced chemokine secretion and prevented early T-cell recruitment in mice lungs after antigen challenge	[29]
**STAT1/3** **dODN**	STAT1/3	DM-induced STAT3-mediated mice model of asthma	reduce airway inflammation and AHR in lungs of mice challenged with HDM	[30]
**C188-9**	STAT3	HDM-induced STAT3-mediated mice model of asthma	Normalization of IL-4, IL-5, IL-13, and IL-17A cytokine levels, as well as prevention of HDM-induced increases in Th2 cells, Th17 cells, and IL-4- and IL-17A-producing non-T cells	[22]
**IBD**				
**Tocilizumab (MRA)**	anti-IL6R MAb	Phase II Clinical: 36 patients with active Crohn’s disease (Crohn’s Disease Activity Index [CDAI] >150) randomly assigned to receive IV infusion of placebo/MRA/alternate MRA-placebo 12 weeks at 8 mg/kg	80% of the patients (8 of 10) given biweekly MRA had a clinical response as compared with 31% of the placebo-treated patients (4 of 13; *P* = 0.019).	[31]
**TJ301 (** **Olamkicept** **)**	IL6R antagonist	Safety and Efficacy of intravenous TJ301 in Participants With Active Ulcerative Colitis	Ongoing Study	[32]
**C326**	IL-6 Inhibitory Avimer protein	Placebo-Controlled, Phase 1, Single and Multiple IV Dose Escalation Study of the Safety, in Adults With Crohn’s Disease	Pharmacokinetics, Pharmacodynamics, and Immunogenicity of C326 in Adults With Crohn’s Disease	[33]
**Vidofludimus**	IL17A, IL17F, and IFN-γ	Open-label uncontrolled entrance study of patients with IBD conducted at 13 study centers in Germany, Bulgaria and Romania	12 weeks treatment phase; 8 out of 14 (57.1%) patients with CD and 6 out of 12 (50.0%) patients with UC were in steroid-free remission (complete responders). Another 4 (28.6%) patients in CD and 5 (41.7%) patients in UC were partial responders. Vidofludimus was well tolerated, with no drug-related serious adverse events.	[34]
**Ustekinumab**	anti-IL12p40 MAb	Double-blind, cross-over trial of the clinical effects of ustekinumab in 104 patients with moderate-to-severe Crohn’s disease (Population 1). Open label trial evaluated the effects of 4 weekly subcutaneous injections or 1 intravenous infusion of ustekinumab in 27 patients who were primary or secondary nonresponders to infliximab (population 2).	In population 1, clinical response rates for the combined groups given ustekinumab and placebo were 53% and 30% (*p* 0.02), respectively at weeks 4 and 6, and 49% and 40% (*p* 0.34), respectively at week 8. In a subgroup of 49 patients who were previously given infliximab (neither primary nor secondary nonresponders), clinical response to ustekinumab was significantly greater than the group given placebo (*p* < 0.05) through week 8. In population 2, the clinical responses at week 8 to subcutaneous and intravenous ustekinumab were 43% and 54%, respectively.	[35,36]
**Janex-1**	Jak3	Murine TNBS-induced colitis model	Attenuation of disease manifestations	[37,38,39]
**Tofacitinib GLPG0634/GS-6034/CP-690550**	Jak3	Phase II Clinical: moderate-to-severe UC	inducing clinical responses and remissions and has been FDA approved as the only nonsteroidal oral treatment that induces remission for moderate-to-severe UC	[40,41]
**Filgotinib**	Jak1	Phase II Clinical: Crohn’s Disease		[42,43]
**Upadacitinib (ABT-494, AbbVie)**	Jak1	Phase II Clinical: Crohn’s Disease, Adult patients with active CD, with a CDAI 220-450, an average daily liquid/soft stool frequency (SF) ≥2.5 or daily abdominal pain (AP) score ≥2.0, and Simplified Endoscopic Score for CD (SES-CD) ≥6 (or ≥4 for those with isolated ileal disease), were randomized 1:1:1:1:1:1 to doubleblind induction therapy with placebo (PBO) or ABT-494 at 3, 6, 12, 24 mg twice daily (BID) or 24 mg once daily (QD) for 16 weeks, followed by blinded extension therapy for 36 weeks	This dose-ranging study demonstrated endoscopic improvement and clinical benefit of ABT-494 as induction therapy in patients with moderate-to-severe refractory CD, and a safety profile as expected with a JAK inhibitor in this population.	[44]
**C188-9**	STAT3	Murine models of DSS-induced UC and TNBS-induced CD	All manifestations of DSS-induced UC and TNBS-induced CD in mice were prevented by C188-9 treatment. C188-9 treatment also induced increased apoptosis of pathogenic CD4^+^ T-cells, and reduced colon levels of IL-17-positive cells in both models.	[45,46]
**Cachexia**				
**STAT3 SH2 domain mimetic peptide (SIP)**	STAT3	C2C12 cell culture model of muscle differentiation	48h treatment resulted in modest myofiber hypertrophy and prevented IL-6-induced fiber atrophy	[47]
**C188-9**	STAT3	CDK cachexia mouse model	C188-9 treatment antagonized catabolic signaling by decreasing myostatin expression and the activation of its downstream signaling mediators, p-Smad2 and p-Smad3. In addition, C188-9 increased muscle mass in tumor-bearing mice by augmenting muscle protein synthesis and suppressing protein degradation	[48]
**AR-42**	HDAC inhibitor	C26 cachexia mouse model	anabolic androgen therapy in combination with HDAC Inhibitor AR-42 was shown to block STAT3 mediated muscle atrophy.	[49]
**Ruxolitinib (INCB018424)**	Jak1/2, STAT3	Incyte, 127 patient, randomized phase II, Cancer associated weight loss trial focused on exocrinemetastatic pancreas cancer patients who had failed first-line chemotherapy, who typically suffer an inexorable decline in weight. Patients received capecitabine and, in addition, were randomly assigned to ruxolitinib versus placebo	The trial’s primary endpoint focused on survival, justified based on the negative prognostic effect of cancer-associated weight loss [11]. The hazard ratio for survival was 0.79 (one-sided *P* ¼ 0.12), but in an a priori subgroup analysis which was intended to identify patients most likely to benefit from JAK inhibition, the hazard ratio for survival was 0.47 (one-sided *P* ¼ 0.005). In this same subgroup, the 6-month survival rate with ruxolitinib was 42% compared to 11% with placebo. Importantly, ruxolitinib-treated patients manifested a significant improvement in body weight compared with placebo. Ruxolitinib was also relatively well tolerated	[50,51]
**Fibrosis**				
**JSI-124**	Jak2	bleomycin-induced lung fibrosis in rats	In rats administered JSI-124, a Jak2 inhibitor that targets STAT3 indirectly, several markers of bleomycin-induced lung fibrosis were reduced	[52,53]
**cucurbitacin-B**	Jak2	carbon tetrachloride (CCl_4_) model of fibrosis	Decreased fibrosis and diminished levels of hydroxyproline in liver tissue as well as expression of collagen-1α, α-SMA and TGF-β	[54]
**Pacritinib (SB1518)**	Jak2	Myelofobrosis, AML (Combined With Decitabine/Cytarabine)	Active drug in myelofibrosis. Going in the AML patients for safety efficacy as a STAT3 inhibitor in combination with Decitabine/Cytarabine	[55,56]
**Ruxolitinib (INCB018424)**	Jak1/2	COMFORT (COntrolled MyeloFibrosis Study with ORal JAK Inhibitor Therapy)-I Trial	Ruxolitinib provided significant reductions in splenomegaly, improvements in myelofibrosis (MF)-related symptoms including cachexia, and a survival advantage relative to placebo in patients with intermediate-2 or high-risk MF. Ruxolitinib treatment was associated with increased weight (mean change: 3.9 kg vs. −1.9 kg), total cholesterol (mean percentage change: 26.4% vs. −3.3%), and albumin levels (mean percentage change: 5.8% vs. −1.7%) at week 24; sustained improvements were observed with longer-term ruxolitinib therapy.	[57,58,59]
**S3I-201**	STAT3	preclinical animal mouse model of renal interstitial fibrosis induced by unilateral ureteral obstruction	Attenuated interstitial fibrosis and showed a fibrotic suppression profile similar to other inhibitors	[60,61]
**C188-9**	STAT3	Bleomycin-induced lung fibrosis Bleomycin-induced and (Tsk-1) models of scleroderma hepatocyte-specific *Pten* knockout (Hep*Pten*-) mouse model of nonalcoholic steatohepatitis (NASH),	C188-9 ameliorated the development of pulmonary fibrosis, reduced expression of genes associated with type II alveolar epithelial cell (AEC) injury and fibrosis, blocked myofibroblast differentiation. Reduced fibrosis in both models of scleroderma as measured by loss of collagen accumulation and decrease in dermal and hypodermal thickness Reduced liver steatosis and hepatic fibrosis in addition to blocking progression of hepatocellular carcinoma	[62,63,64,65]

S1PR2: sphingosine-1 phosphate (S1P) receptor 2 (R2), NA: Not available, MAb: Monoclonal Antibody, SM: small molecule, pY: STAT3 phosphorylation at Tyr-705.
